# Rapid semi-automated quantitative multiplex tandem PCR (MT-PCR) assays for the differential diagnosis of influenza-like illness

**DOI:** 10.1186/1471-2334-10-113

**Published:** 2010-05-11

**Authors:** Elektra Szewczuk, Kiran Thapa, Terry Anninos, Kenneth McPhie, Geoff Higgins, Dominic E Dwyer, Keith K Stanley, Jonathan R Iredell

**Affiliations:** 1 AusDiagnostics Pty Ltd, 3/36 O'Riordan Street, Alexandria, NSW 2015, Australia; 2 Centre for Infectious Diseases and Microbiology, University of Sydney, Westmead Hospital, Westmead NSW 2145, Australia; 3 Institute for Clinical Pathology and Medical Research, Westmead Hospital Westmead NSW 2145, Australia; 4 Institute of Medical and Veterinary Science, Frome Road, Adelaide, SA 5000; Australia

## Abstract

**Background:**

Influenza A, including avian influenza, is a major public health threat in developed and developing countries. Rapid and accurate detection is a key component of strategies to contain spread of infection, and the efficient diagnosis of influenza-like-illness is essential to protect health infrastructure in the event of a major influenza outbreak.

**Methods:**

We developed a multiplexed PCR (MT-PCR) assay for the simultaneous diagnosis of respiratory viruses causing influenza-like illness, including the specific recognition of influenza A haemagglutinin subtypes H1, H3, and H5. We tested several hundred clinical specimens in two diagnostic reference laboratories and compared the results with standard techniques.

**Results:**

The sensitivity and specificity of these assays was higher than individual assays based on direct antigen detection and standard PCR against a range of control templates and in several hundred clinical specimens. The MT-PCR assays provided differential diagnoses as well as potentially useful quantitation of virus in clinical samples.

**Conclusions:**

MT-PCR is a potentially powerful tool for the differential diagnosis of influenza-like illness in the clinical diagnostic laboratory.

## Background

Approximately one billion cases of influenza occur around the world every year, despite the availability of effective vaccines. These are associated with an estimated 3-500 000 deaths and 3-5 million hospitalizations annually and impose a significant economic burden on even the most developed countries [1,2].

In an epidemic or pandemic setting, the rapid differentiation of influenza A from other influenza-like illness (ILI) is essential for infection control and patient management [3]. However, a relatively non-specific presentation means that influenza cannot be reliably distinguished from other ILI presenting in the autumn and winter seasons, and currently available diagnostic systems lack both the sensitivity and specificity required for efficient differentiation of ILI at the health facility or triage station [4]. A variety of different diagnostic tests are available to detect most common respiratory viruses, but there is still an unmet need for rapid, sensitive and accurate diagnosis. Commonly used conventional methods of virus culture, serological testing or antigen detection by direct immunofluorescence are reasonably sensitive, but relatively complex and labour-intensive, and are generally too slow to be clinically relevant [5]. Rapid antigen or "point-of-care" tests are widely available for influenza and RSV [6-8] and take only about 15-30 minutes to perform, but their sensitivity is lower than nucleic acid testing (NAT)[9,10].

Predictable oseltamivir resistance occurring within outbreaks of certain influenza subtypes and the presence of several co-circulating influenza A viruses of different epidemic potential also makes subtyping particularly valuable and this is not generally available in any of the rapid methods. Here, we used the recently described MT-PCR method [11] to develop quantitative assays for the most important causes of ILI and the identification of Influenza A subtypes, and evaluate their performance in two clinical laboratories.

## Methods/Results

### Clinical and control specimens

Combined nose and throat swabs collected at the Centre for Infectious Diseases and Microbiology (CIDM, at Westmead Hospital in Sydney) in the southern hemisphere winter of 2006 and 2007 were used for initial assay validation at the Centre For Immunology at St Vincents Hospital in Sydney (CFI). Gamma-irradiated MDCK-tissue culture extracts with known amounts of Indonesian (Indo 05, Indonesia "clade 2") and Vietnamese (HN 3028, Vietnam "clade 1") strains of H5N1 influenza A virus were provided Australia-wide as control templates [12], and we used these along with locally cultivated tissue culture extracts of H3N2 A/Fujian/114/2002-like and H1N1 A/New Caledonia/20/99-like Influenza A virus and B/Shanghai/361/2002 Influenza B virus.

### Immunofluorescence

This was performed using fluorescein-conjugated monoclonal antibodies (Chemicon International, Temecula, CA, USA) against influenza A and B, RSV, adenoviruses, and hPIV1-3 on acetone-fixed smears of material from respiratory tract specimens, as indicated and as previously described [13].

### DNA handling and sequencing

Nucleic acid was extracted from clinical specimens and from tissue culture using the High Pure Viral RNA Extraction kit (Roche Diagnostics GmbH, Mannheim, Germany), eluting 200 μl of sample into 50 μl of elution buffer. A negative control was extracted with every batch. MT-PCR primers were designed with a modified version of Primer 3 software [14], and were based on alignments of available sequences deposited in GenBank (Table 1) and of cDNA amplicons obtained from known virus isolates at CIDM cloned into pGEM-T (Promega) as positive controls. A nested reverse transcriptase polymerase chain reaction (RT-PCR) was used to detect influenza A and B and other respiratory viruses, as previously described [13,15,16]. DNA sequences of all PCR amplicons were determined as needed by cloning into pGEM-T for M13 dye primer sequencing (Promega) and were compared with known sequences using EclustalW (ANGIS). The melt temperature, with a range of 1.5°C either side of predicted Tm, was deemed acceptable if the melt curve was normal sigmoidal. Verification of correct-sized discrete amplicons derived in step 2 MT-PCR assays was performed using a Bioanalyzer DNA separation chip (Agilent Technologies, Forest Hill, Victoria, Australia), as previously described [11].

**Table 1:** Gene targets for MT-PCR respiratory virus profile 1.

assay	gene target	Example reference sequences^a^	
INF-A	Influenza A nucleoprotein	A/New York/392/2004(H3N2) [GenBank NC_007366-73]; A/Sydney/05/97(H3N2) [GenBank CY039082]; A/Puerto Rico/8/34(H1N1) [GenBank NC_002016-23]; A/New Caledonia/20/99(H1N1) [GenBank AJ458265]; A/New Zealand/7/1983(H1N1) [GenBank CY020189-196]; A/Indonesia/7/2005(H5N1) [GenBank EU146637]; A/Indonesia/175H/2005(H5N1) [GenBank EU146643]; A/Viet Nam/1203/2004(H5N1) [GenBank AY818038]; A/Viet Nam/1194/2004(H5N1) [GenBank AY651498];	
INF-B	Influenza B nucleoprotein	B/lee/40 [GenBank NC_002208.1]	
H1	Influenza A haemagglutinin	A/Puerto Rico/8/34(H1N1) [GenBank NC_002016-23]; A/New Caledonia/20/99(H1N1) [GenBank AJ344014] A/New Zealand/7/1983(H1N1) [GenBank CY020189-196]; A/Beijing/262/95(H1N1) [GenBank EF541421];	
H3	Influenza A haemagglutinin	A/Sydney/5/1997(H3N2) [GenBank CY039079]; A/Fiji/185/2004(H3N2) [GenBank EF566069/EH5041403]; A/New York/392/2004(H3N2) [GenBank NC_007366-73]; A/Aichi/2/68(H3N2) [GenBank AB295605]	
H5^b^	Influenza A haemagglutinin	A/Viet Nam/LA-028/2005(H5N1) [GenBank DQ099782]^c^; A/Viet Nam/1203/2004(H5N1) [GenBank AY818135]^c^; A/Viet Nam/JP14/2005(H5N1) [GenBank EF456799]^c^; A/Viet Nam/1194/2004(H5N1) [GenBank EF551402]^c^; A/Indonesia/7/2005(H5N1) [GenBank EU146632]^d^; A/Indonesia/175H/2005(H5N1) [GenBank EU146640]^d^; A/Indonesia/5/05(H5N1) [GenBank EF541394]^d^;	
RSV	Human Respiratory Syncytial Virus polymerase (L)		[GenBank NC_001781.1] (nt8509-15009)
hPIV-3^e^	Human Parainfluenza virus type 3 nucleocapsid		[GenBank NC_001796.2] (nt111-1658)
RV	Human Rhinovirus 5' untranscribed region (5'-UTR)		[GenBank EU700020-28]

^a^NCBI reference sequences (NC_) cited where possible.

^b^Influenza profile 3 does not include H5; ^c ^"clade 1" virus; ^d ^"clade 2" virus;

^e^Influenza profile 1 does not include hPIV-3

### Multiplexed tandem PCR (MT-PCR)

**Figure 1:**
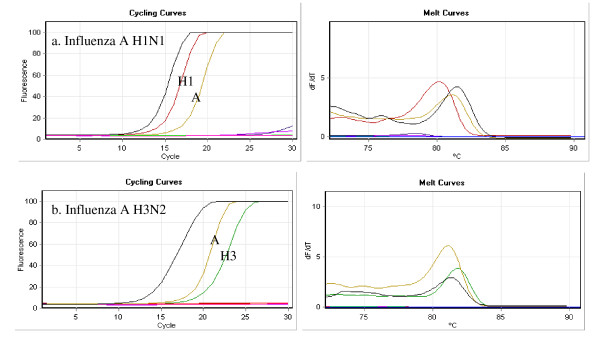
Cycling and melt curves for Influenza A H1 and H3

This is a two-step assay using nested primer pairs in which the first step involves a highly multiplexed reaction to pre-amplify multiple targets for (typically, 15) cycles. These are then aliquoted into individual reaction tubes containing nested specific PCR primers as template for the second step reaction [11] which was performed using a liquid handling robotics system provided by AusDiagnostics Pty Ltd. (Sydney, Australia). Primer and artificial internal control sequences are not shown due to commercial confidentiality agreements with AusDiagnostics Pty Ltd. The target regions are specified in Table 1, with example sequences. The internal control is a contrived sequence that does not appear in nature. No base pairing redundancies were specified in any of the primers used. Lyophilized primers and reagent kits were prepared at CFI and sent to CIDM, and to the Institute of Medical and Veterinary Science (IMVS, at Royal Adelaide Hospital in Adelaide), for further testing of clinical samples collected during routine investigation of ILI outbreaks and sporadic infection at these two referral centres. For MT-PCR performance, a strip tube containing Step 1 multiplexed primers was placed in a thermal cycler, along with a Gene-Disc containing lyophilised Step 2 reagents and oil (for covering PCR reactions) on the robot deck. The samples were directly added to the strip tube in the thermal cycler. A software template for the reaction was then selected and all operations for performing the Step 1 multiplexed preamplification, dilution and aliquoting into the Step 2 reaction tubes in the Gene-Disc were performed automatically. The Gene-Disc was then hermetically sealed in a heat-sealer and Step 2 amplification carried out in a Rotor-Gene RG6000 thermal cycler. At the end of Step 2 the presence or absence of each target was automatically called using a software routine (AusDiagnostics Pty Ltd.) that compared melt temperature of the product with expected values and checked the purity and quantity against predetermined threshold values, and these were all manually verified. The cycle threshold (Ct) is the number of cycles at the takeoff point of the amplification curve (see Figure 1). Quantitation was by comparison with the internal control, which was assigned an arbitrary value of 10 000, chosen so that the lowest concentration target detected had a value above "1". Concentration of the final step 2 product was expressed relative to the control as previously described [11].

### Assay development

Assays were developed to detect Influenza viruses A and B (INF-A and INF-B), the Respiratory Syncytial Virus (RSV), Rhinovirus (RV), and human Parainfluenza virus type 3 (hPIV-3), and to identify Influenza A haemagglutinin gene types H1 (not H1N1/09), H3 and H5 within a multiplexed tandem PCR (MT-PCR) assay profile [11], using sequences available from strains in our laboratory and lodged in GenBank, including the reference sequences listed in Table 1. Multiplexed assays are generally configured so as to divide neatly into 72 (the number of positions available in a standard step 2 template in the RG6000 cycler), with no empty wells remaining. Primers targeting the Influenza A haemagglutinin H5 gene (in "Influenza profile 1") were replaced by primers targeting the nucleoprotein gene of human Parainfluenza virus type 3 (hPIV-3) in another assay ("profile 3"). All data were deidentified; additional testing of laboratory samples was approved as a Quality Assurance activity by the Sydney West Area Health Service Human Research Ethics Committee

### Specific identification of influenza and RSV in positive controls

**Figure 2:**
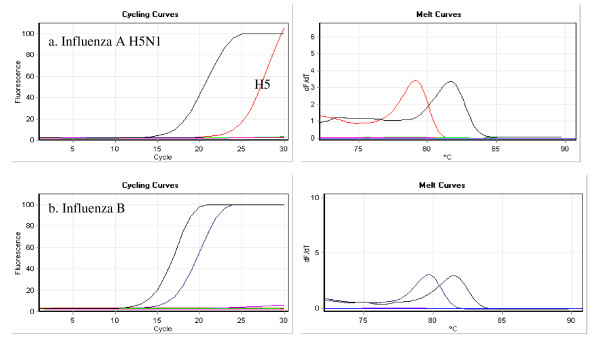
Cycling and melt curves for Influenza A H5 and Influenza B

**Figure 3:**
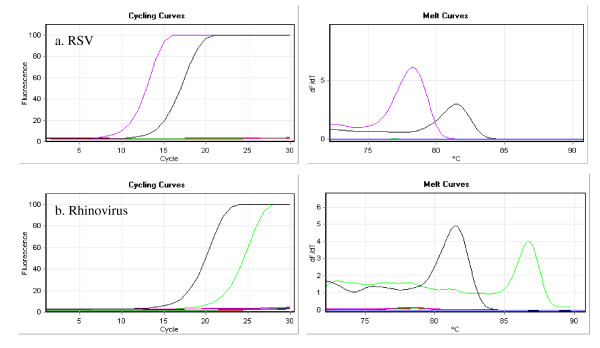
Cycling and melt curves for Respiratory Syncytial Virus and Rhinovirus

**Figure 4:**
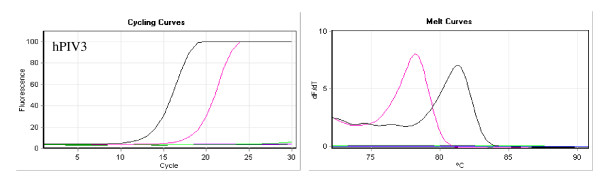
Cycling and melt curves for Parainfluenza virus type 3

Thirty-nine viral cell-culture extracts from stored clinical isolates (including isolates identified as INF-A n = 5, INF-B n = 2, RSV n = 1, RV n = 7, along with undifferentiated picornavirus n = 4, and one each of hPIV-1, hPIV-3, coronavirus 229E, coronavirus OC43, hMPV, enterovirus EV-68, adenovirus type 3 and adenovirus type 4) were tested using Influenza Profile 1 (INF-A, INF-B, RV, RSV, H1, H3, H5) in blinded fashion in the lab at which the assays were developed (CFI). Signals were all normalised against an internal control value of 10 000 arbitrary units. All INF-B and RSV were detected only in the known control samples and no positive signals for H5 were observed in any sample nor in (water-only) controls, in triplicate assays (data not shown). Detection of INF-A was 100% sensitive and specific. Example (step 2) cycle and melt curves are shown in Figures 1, 2, 3, 4, after 15 pre-amplification cycles in Step 1.  Step 2 cycling and melt curves in multiplex assays of gene targets specific for influenza A, influenza B, respiratory syncytial virus (RSV), rhinovirus (RV) and human para-influenza virus 3 (hPIV3) in Figures 1-5. In all panels, right and left colour schemes correspond; black line: internal control; A: INF-A.

### Specific detection of Influenza A haemagglutinin subtypes

To specifically examine INF-A subtype detection, we tested serially diluted irradiated whole extracts and cell culture supernatants of reference strains of H1N1, H3N2, and Indonesian and Vietnamese H5N1 strains with Influenza Profile 1 (Table 1), with the most diluted samples containing only 1-10 copies of RNA virus/μl. Where one haemagglutinin type gave a greater than 100-fold fluorescence signal than another after normalization against the internal control, it was deemed the correct result.

Due to limited availability of control samples, only 2 μl of eluate (equivalent to the same concentration of virus if extraction efficiency was 100%) was used for analysis (5 μl is recommended for extracted nasal pharyngeal samples) and all assays in Table 2 were performed only once. Serial ten-fold dilutions of H1 whole cell culture extracts from 10^-1 ^down to 10^-4 ^yielded specific signals for H1 (63904 down to 188 normalised units) and INF-A (67191 down to 231) only. Similarly, serial dilutions of H3 whole cell culture extracts from 10^-1 ^down to 10^-4 ^yielded specific signals for H3 (33064 to 295) and INF-A (4734 to 15), with only very low cross-reactivity with H1 in the least diluted samples but with a 500-fold differential (e.g. H3 = 33064 and H1 = 66 at 10^-1^). H5-specific signal was obtained from all but the highest dilutions (10^-6^) of whole cell culture extracts, in which INF-A was detected at low level. Low-level cross-reactivity (less than 1:200 relative to H5) was observed with H1 (Table 2). The concentration of virus in H5N1 samples (shown in parentheses in Table 2) had been previously determined [12] and were detected at levels as low as a single copy of virus/μl in the original sample in both strains.

**Table 2:** Specific detection of haemagglutinin subtypes.

specimen	H1	H3	H5	INF-A	result
H1N1 (A/New Caledonia/20/99-like)					
Cells 10^-1^	^a^63904	-	-	67191	H1
Cells 10^-2^	4856	-	-	8362	H1
Cells 10^-3^	2492	-	-	3023	H1
Cells 10^-4^	188	-	-	231	H1

H3N2 (A/Fijian/114/2002-like)					
Cells 10^-1^	66	33064	-	4734	H3
Cells 10^-2^	-	6456	-	931	H3
Cells 10^-3^	-	1570	-	142	H3
Cells 10^-4^	-	295	-	15	H3

H5N1 (HN 3028 Vietnam; clade 1)					
S/N 10^-2^	-	-	97528	7	H5
S/N 10^-3^	1	-	865	3	H5
S/N 10^-4^	27	-	8490	25	H5
S/N 10^-5^	-	-	-	12	(INF-A)
Cells 10^-1 ^(10^4^/μl)	2	-	133177	1	H5
Cells 10^-2 ^(10^3^/μl)	5	-	116490	2	H5
Cells 10^-3 ^(10^2^/μl)	2	-	49011	19	H5
Cells 10^-4 ^(10^1^/μl)	38	-	11836	128	H5

H5N1 (Indo 05 Indonesia; clade 2)					
S/N 10^-1^	-	-	2489082	-	H5
S/N 10^-2^	30	-	167289	6	H5
S/N 10^-3^	4	-	143625	6	H5
S/N 10^-4^	-	-	156819	5	H5
Cells 10^-1 ^(10^4^/μl)^b^	-	-	522949	-	H5
Cells 10^-2 ^(10^3^/μl)	-	-	64783	6	H5
Cells 10^-3 ^(10^2^/μl)	37	-	194809	1431	H5
Cells 10^-4 ^(10^1^/μl)	72	-	16788	9	H5
Cells 10^-5 ^(10^0^/μl)	19		249234	7055	H5
Cells 10^-6 ^(10^-1^/μl)	9	-	-	234	(INF-A)

^a ^values obtained after normalisation against internal control;

^b ^copies of virus per μl in each original sample [12]

### Testing of MT-PCR assays with known clinical specimens

We next tested the same set of assays (Influenza Profile 1) at CIDM on 5 ul of the nucleic acid extracts obtained from forty-five clinical specimens retrieved from storage at -70 deg C, all of which had previously been collected during investigation of influenza in an institution. Several subjects had been taking the antiviral agent oseltamivir at time of sampling. Some of these specimens had been tested by IF, some by PCR and some by both methods.

All of 18 previously identified INF-A samples were recognised by the MT-PCR INF-A assay, normalised values ranging from 9 to 68590. All of these were retrieved from long-term storage, and template degradation was a significant possibility. Six of these could not be assigned a specific haemagglutinin subtype, and most of these latter (5/6) had a normalised INF-A value < 30. All three known INF-B samples and all eight RSV-positive samples were correctly identified by MT-PCR, with normalised values of 136-2379 and 4306-264304, respectively (data not shown).

Thus, MT-PCR recognised all of the known positive INF-A samples; previous RT-PCR on these extracts and IF results on original samples were all congruent where both had been performed (Table 3). However, six of the sixteen samples reported negative by IF for INF-A were positive by MT-PCR (not shown). Of these six, one was also weakly positive by RT-PCR for INF-A (but had been reported as a negative result) and earlier specimens from the same patients had been positive for INF-A in another four cases. These additional INF-A detections were all low-level (normalised result < 100) but all had correct-sized discrete second-stage amplicons as determined on Bioanalyzer (Agilent, SantaClara, CA) with sigmoidal cycling curves and single melt peaks and are thought to represent true positive results. Sequencing was performed on all six of the unexpected positives to confirm the results.

**Table 3:** Comparison of IF and PCR with MT-PCR Influenza profile 1.

	previous result	MT-PCR result^a^	
**IF (total)**	**32**		

INF-A (unspecified)	6	6	(H3 n = 4; H1 n = 1)^b^
RSV	8	8	
total INF-A/RSV	14	22	(14 + INF-A n = 8)^c^
total negative	18	10	

**PCR (total)**	**37**		

INF-A (unspecified)	17	17	(H3 n = 5; H1 n = 1)
INF-B	3	3	
RSV	2	2	
total INF-A/-B/RSV	23	28	(+ INF-A n = 5)
total negative	14	9	

**IF or PCR (total)**	**45**		

all INF-A	18	24	
all INF-B	3	3	
all RSV	8	8	
all positive results	29	35	
all negative results	16	10	

^a^profile 1 includes assays for INF-A, INF-B, H1, H3, H5, RSV and RV but not hPIV3; ^b^all 6 were also PCR positive; ^c^not including INF-B (n = 3) detected by MT-PCR in samples which had not been tested for these pathogens

Of those that tested positive by MT-PCR, IF had detected a significant pathogen in 14/22 (64%) and in-house RT-PCR had previously detected 23/28 (82%).

### Comparison of MT-PCR assays with immunofluorescence

For greater clinical utility in the Australian influenza season, we then replaced the H5 assay with an hPIV-3 assay (Influenza profile 3: INF-A, INF-B, H1, H3, RSV, RV, hPIV-3) and tested this profile independently in two clinical laboratories (CIDM and IMVS) (Table 4).

**Table 4:** Comparison of IF and PCR with MT-PCR Influenza profile 3.

	previous result	MT-PCR result^a^	
**IF CIDM (total)**	**276**		

INF-A (unspecified)	38	35	(H3 n = 28)
INF-B	1	1	
RSV	15	15	
hPIV-3	3	3	
total +ve	56	73	(53 +INF-A n = 14, INF-B n = 1, RSV n = 5)
-ve	220	^b^203	

**PCR IMVS (total)**	**176**		

INF-A (unspecified)	41	41	(H3 n = 27; H1 n = 9)
INF-B	13	13	
RSV	22	22	
hPIV-3	11	11	
total +ve	87	88	(+INF-A n = 1)^c^
-ve	89	85	

**IF or PCR (total)**	**452**		

all INF-A	79	90	(3 IF +ves for INF-A not detected)
all INF-B	14	14	
all RSV	37	37	
all hPIV-3	14	14	

all positive results	144	156	(+INF-A n = 15)

all negative results	308	^b^296	

^a^profile 3 includes assays for INF-A, INF-B, H1, H3, RSV, RV and hPIV3 but not H5; ^b^not including additional hPIV-3 (n = 2) or RSV (n = 10) detected by MT-PCR in samples which had not been tested for these pathogens; ^c^not including RSV/INF-A and RV/RSV co-infections

Two hundred and seventy-six clinical specimens tested by IF at the CIDM were tested by MT-PCR; congruent results were obtained in 53/56 IF-positive specimens; three of the 38 INF-A specimens were not detected by MT-PCR. Two of these three could not be confirmed by RT-PCR for INF-A either, suggesting a false-positive IF result (no inhibition was detected).

220 additional specimens were negative by IF: 14 of these samples were also positive by MT-PCR for INF-A (and/or H3), one for INF-B, and five for RSV (these five all coming from symptomatic patients taken during investigation of an RSV outbreak). All of these 14 additional positive MT-PCR results were low-level (< 50 normalised units). Two additional hPIV-3 and ten RSV were also detected in samples that had only been tested for INF-A and INF-B, but these are not included in Table 4.

### Comparison of MT-PCR assays with standard RT-PCR

One hundred and seventy-six additional clinical specimens, completely unrelated to those tested above, were tested by RT-PCR at the IMVS [16]: 87 were reported positive and MT-PCR results were congruent in all of these (Table 4). Of the 89 negative results, five tested positive by MT-PCR (profile 3): one was a very low-level INF-A, while the other four were RSV and RV. These specimens had not previously been tested for these pathogens and the MT-PCR results are not included in Table 4. An INF-A/RSV co-infection which had been already identified by RT-PCR was identified as an INF-A H3/RSV co-infection by MT-PCR (not shown).

### Relative quantities of influenza virus in clinical specimens

The quantitative nature of MT-PCR potentially allows the comparison of normalised values over time but serial INF-A values alone may be misleading if the number of cells in the sample (a measure of sample "quality") varies significantly. Expressing the INF-A value as a function of the normalised value of the human NONO (non-POU domain containing, octamer-binding) gene (GenBank Accession No. NC_000023.9) gives an approximation of the amount of human epithelial cells harvested in sampling. INF-A/NONO ratios derived from a small subset of patients were assayed using the assays in Influenza profile 4 (INF-A, INF-B, H1, H3, H5, RSV, RV, hPIV3 and NONO) (Table 5). The set of INF-A/NONO ratios in Table 5 were derived from assays of serial samples of patients treated with oseltamivir for influenza A during the course of management of an institutional outbreak, demonstrating the potential for quantitation as a marker of sample quality (normalised NONO value) and perhaps of the course of infection (INF-A/NONO ratio).

**Table 5:** Changing INF-A/NONO ratios in treated cases.

#	day 0	day 3	day 6
**a**	6.7	92.8	61.9
**b**	139.8	43.3	6.4
**c**	900.0	666.7	0
**d**	21.2	4.2	0
**e**	26523.8	90.9	0
**f**	26.7	315.3	0
**g**	200.7	9.61	0
**h**	335.8	0.3	0
**i**	6854.5	5.5	3.5
**j**	6333.3	126.4	1.9
**k**	187071.4	52.6	1.2
**l**	6854.5	5.5	3.4

normalised ratios are shown, from day 0 (first contact with patient) to day 6; individual cases identified only as alphabetical letters.

## Discussion

Detection methods are available for the major ILI pathogens (Table 1), as well as for the 15 known H and 9 known N variants [17-22]. Antigen-based rapid assays are generally quite specific but range from 60-70% in sensitivity [6,8]; PCR methods appear to be the most sensitive and specific, with detection limits down to < 0.1 TCID_50_/mL [23-26]. Multiplex PCR must deal with the competition and interference that arises as a result of using large numbers of primers simultaneously. DNA hybridisation after multiplexed PCR is one solution but is slow and labour-intensive [27,28]. MT-PCR deals with the problem by stopping the multiplexed first round of amplification before competition develops, then using the amplified products as template for a second round of individual specific 'nested' reactions [11].

This evaluation does not control for variation in specimen quality or operator performance or any of a number of other variables applicable in real working laboratories. We used an automated liquid-handling system and robotic cycler that operates on a preset algorithm for both cycling and for calling of results. All results were manually verified in this study but required operator expertise is otherwise minimal. Second-stage amplified product is sealed and discarded unopened. Every second-step 72-well gene disc has 12 wells for each target of a 6-target assay, all separated spatially. The additional positives we describe came on different days, all with negative controls and with several other negatives in the runs in which they were detected. All MT-PCR reactions yielded normal melt curves and all reactions giving unexpected results also contained discrete correct-sized amplicons. No multiple or unexpected-size amplicons or discrepant melt temperatures were identified. Nevertheless, sequencing of clinical isolates was not performed after initial test validation and no additional alternative independent testing was performed to specifically verify second-step results, so contamination of the second-step reaction with unrelated first-step amplicons cannot be completely excluded.

The MT-PCR method is essentially a nested RT-PCR and would therefore be expected to be more sensitive than a conventional PCR or direct antigen detection system [29]. Specificity may thus be underestimated if MT-PCR detects target sequences in material below levels detected by conventional methods. Testing of control templates gave "clean" results with no evident cross-reaction (Figures 1, 2, 3, 4) and the unexpected positive results obtained in "negative" clinical specimens were almost uniformly at low levels, consistent with this as an explanation. Also consistent, unexpected additional positive results from MT-PCR were much more common in specimens deemed negative by IF than by PCR (see Table 4). For example, the additional MT-PCR-positive IF-negative samples at CIDM (n = 14) for INF-A were collected from symptomatic patients during an influenza outbreak and several were either confirmed by RT-PCR or were associated with seroconversion (greater than four-fold increase) or initial high (> = 32) titres to Influenza A. This level of additional positive results (~10%) is very similar to that seen in previous reports for assays of this type [30]. Taken all together and including assays tested within both profiles, MT-PCR correctly identified 141 (97.9%) of 144 clinical specimens found positive by standard methods. Conversely, taking into account all of those specimens found to be positive by MT-PCR in this study, IF detected a significant pathogen in 56/73 (77%) and in-house RT-PCRs detected 87/88 (99%). For influenza A detection, this was a relative sensitivity of 38/49 (78%) for IF and 41/42 (98%) for RT-PCR [16], compared to MT-PCR.

IF and MT-PCR were entirely congruent in clinical specimens tested for INF-B (n = 17), RSV (n = 44) and hPIV-3 (n = 14). Of the three samples positive by IF but negative by MT-PCR for INF-A, only one could be confirmed on repeat testing. If these false-positive IF results are set aside, the MT-PCR sensitivity relative to any reference laboratory positive result would be recalculated as 141/142 (99.3%) and, if the 47 positive results obtained from the 39 cell cultures and 22 additional controls in Table 2 included, as 188/189 (99.5%). Conversely, the IF detected 70/95 (74%) and RT-PCR detected 110/116 (95%) of those that were MT-PCR positive for any pathogen, which included many additional low-level positives. For INF-A, the relative sensitivity overall of IF relative to MT-PCR was 44/63 (70%) and of in-house RT-PCR was 58/64 (91%).

Human Influenza virus replicates throughout the respiratory tract and viral loads normally peak (10^3 ^to 10^7 ^TCID_50_/mL of nasopharyngeal samples) at 24-72 hours after onset of symptoms. Determining the infectious period and its variability is crucial for individual and public health planning but are likely to be both host- and strain-specific [24,31,32]. In previous testing of RNA targets using MT-PCR, the correlation of quantitation over two logs of template concentration was 0.99 and the coefficient of variation over ten independent cycle threshold (Ct) measurements was 0.03 [11]. Here, we also used NONO as a marker of human epithelial cells in the specimen to normalise viral titres (Table 5). For well-collected specimens with significant amounts of human epithelial cells present, the INF-A/NONO ratio may be useful to monitor viral shedding and infectivity.

However, actual primer sequences may fail to recognise new viral variants and so a lack of transparency by manufacturers in releasing such information requires evidence that the very latest sequences available are completely homologous with the primers included, if clinical laboratories are to trust the assays. Given the ready availability of influenza A sequences (eg. via PubMed), it is reasonable to expect this as a minimum standard. Our testing of fully-characterised strains included only those listed in Table 2, and the reference sequences used are listed in Table 1. In this study, the isolates tested in the clinical laboratories came only from two Australian population centres and different regions may encounter strains which are significantly different from those listed in Tables 1 and 2.

The initial H5 target is a consensus for clades 1-3 of the highly pathogenic H5N1 strains, consistent with WHO guidelines http://www.who.int/csr/disease/avian_influenza/guidelines/RecAIlabtestsAug07.pdf, but the region targeted by our consensus INF-A primers varies significantly from that of the highly pathogenic avian strains of Influenza A, and this was reflected in a relatively low sensitivity in the INF-A assay for H5N1. Since this assay was developed, we have also experienced the influenza A/H1N1/09 pandemic, which caused a significant number of intensive care admissions and deaths in this country (33) and required modifications to include the NP gene of A/H1N1/09 and an M gene consensus (H1N1; A/H1N1/09; H3N2; H5N1). Performance in the Australian 2009 influenza season appears comparable to a nested RT-PCR specifically optimised for A/H1N1/09 only [9,10].

The diagnosis of co-infections (e.g. RSV and INF-A) and other causes of ILI may be clinically important. Pandemic influenza dramatically increases requirements for high-level Intensive Care [33] and the ability to efficiently distinguish true INF-A from ILI of a less threatening nature in the context of an outbreak or epidemic may be vital to minimise the burden on the health infrastructure [34]. Respiratory syncytial virus (RSV) is the single most important cause of acute lower respiratory tract diseases including bronchiolitis and pneumonia in infants and young children and may contribute to hundreds of thousands of deaths annually among the elderly and immunocompromised [1,35,36]. Human Parainfluenza virus type 3 (hPIV-3) is associated with pharyngitis, bronchiolitis and pneumonia in children, and respiratory infections in adults and is the second most significant acute viral respiratory tract infection in young children after RSV. Both are leading causes of hospitalization in adults with acute community-acquired respiratory disease. The rhinoviruses (RV), of which there are more than 100 serotypes, act either as single pathogens or in mixed infections, but all of these may present as a non-specific ILI.

## Conclusions

We have tested several hundred clinical samples in two reference laboratories and found multiplexed tandem (MT)-PCR to be comparable to target-specific RT-PCR and a great deal more sensitive than direct immunofluorescence for the detection of several of the most important causative agents of influenza-like illness. Importantly, this method simultaneously subtypes Influenza A, identifies multiple pathogens and co-infections in a single specimen and provides potentially valuable quantitative data. A multiplexed method also facilitates recognition of co-circulating viruses in outbreak situations in which they might otherwise be assumed identical. It is clear that, as for influenza vaccine strategies, primer designs need to be reviewed regularly in light of prevalent sequence variations and the configuration of the multiplexed assay itself needs to be informed by the relevant epidemiology of the region. The assay we describe presently costs around AUD$15 (USD$12) for 6 targets (~USD$2 per target). Even with additional purification and labour costs, this is potentially cost-effective in the routine laboratory.

## Competing interests

KS developed and patented MT-PCR and is the principal of AusDiagnostics, which now commercialises these assays. ES was an employee of AusDiagnostics during the course of this research.

## Authors' contributions

ES and KT contributed equally to the paper, conducting the MT-PCR assays in Sydney and preparing raw data for review by KS, DD, and JI. KM, KT, and TA performed other diagnostic assays. TA performed the MT-PCR assays in Adelaide and prepared the data in conjunction with GH. DD, GH, JI advised on assay configuration and targeting. DD obtained crucial control samples (H5). KS developed the target primers, troubleshot the assay with ES, and supervised initial assay development prior to clinical validation studies. KS and ES conducted several diagnostic assay runs in initial development stages. JI conceived the project, obtained the funding, oversaw the assays in CIDM in conjunction with KM and DD, analysed the data with KS, and wrote the paper in conjunction with KS, ES, KT and DD. All authors have read and approved the final manuscript.

## Pre-publication history

The pre-publication history for this paper can be accessed here:

http://www.biomedcentral.com/1471-2334/10/113/prepub
